# Photoprotective and Heavy Metal-Detoxifying Melanin Fractions Isolated from *Ophiocordyceps sinensis* Fermentation Products

**DOI:** 10.3390/biology15141183

**Published:** 2026-07-17

**Authors:** Xiangxin Li, Huan Yang, Yiming Wang, Chuanyong Li, Yanli Huo, Jianzhao Qi, Li He

**Affiliations:** 1 College of Biology and Pharmaceutical Engineering, Lanzhou Jiaotong University, Lanzhou 730070, China; lixx@mail.lzjtu.cn (X.L.); yanghuan001103@163.com (H.Y.); wym030210@163.com (Y.W.); lichuanyong2001@163.com (C.L.); huoylsw@126.com (Y.H.); 2 Center of Edible Fungi, Northwest A&F University, Yangling, Xianyang 712100, China; qjz@nwafu.edu.cn

**Keywords:** *Ophiocordyceps sinensis*, melanin, photoprotective activity, heavy metal relief ability, antioxidant activity

## Abstract

Melanin is a natural biopolymer with broad biological activities, yet research on melanin from the medicinal fungus *Ophiocordyceps sinensis* remains insufficient. In this study, two melanin fractions, TZ-a and TZ-b, were isolated from its fermentation products and characterized by Fourier Transform Infrared Spectroscopy (FT-IR), Scanning Electron Microscopy (SEM), Thermogravimetric Analysis (TGA) and Ultraviolet–Visible spectra (UV-Vis). Their protective effects against UVB-induced damage in keratinocytes and heavy metal (Cd^2+^, Pb^2+^, As^3+^, Hg^2+^) cytotoxicity were evaluated. Both fractions exhibited characteristic melanin peaks but showed structural differences. TZ-a displayed lamellar or needle-like crystals, whereas TZ-b formed block-like aggregates. Both demonstrated good thermal stability. UV-Vis spectra confirmed their typical melanin identity. At 200 μg/mL, TZ-a and TZ-b increased cell viability from ~50% (model) to 80.59% and 82.48%, respectively. Additionally, they elevated the proportion of viable cells within the apoptotic population from 59.11% to 86.08% and 84.79%. Both fractions concentration-dependently alleviated heavy metal-induced cytotoxicity, reduced malondialdehyde levels, and restored glutathione pools. In conclusion, the melanin fractions TZ-a and TZ-b from *O. sinensis* exhibited photoprotective effects and alleviated heavy metal toxicity.

## Simple Summary

*Ophiocordyceps sinensis* is a prized traditional medicinal resource. In this study, we extracted two natural melanin fractions from its fermentation products. We found that both pigments effectively mitigated the damaging effects of UVB radiation on NHEK cells, boosting cell survival rates to over 80%. Additionally, they protected cells against four common toxic heavy metals—cadmium, lead, arsenic, and mercury—by alleviating harmful oxidative stress and restoring intracellular antioxidant reserves. These findings suggest that these natural fungal pigments hold promise as safe and effective ingredients for sunscreens, anti-aging skincare products, and formulations designed to combat environmental pollution.

## 1. Introduction

Melanin is a complex polymer formed by the oxidative polymerization of phenolic or indolic monomers and is widely distributed in animals, plants, and microorganisms [1,2,3,4]. *Ophiocordyceps sinensis*, a rare medicinal fungus traditionally used to nourish the lung and kidney and to enhance immunity, has been shown to contain various active components such as polysaccharides and cordycepin [5,6,7]; however, research on its melanin remains limited. Dong and Yao first isolated melanin from the fermentation broth of *O. sinensis* and confirmed its DPPH radical scavenging and Fe^2+^ chelating activities [8], yet the structural heterogeneity and broader biological activities of this melanin have not been thoroughly investigated.

The abundant functional groups in melanin, including hydroxyl, carboxyl, amino, and phenolic hydroxyl groups, endow it with antioxidant, anti-radiation, and heavy metal chelating properties [9,10]. Ultraviolet B (UVB, 280–320 nm) radiation induces excessive production of intracellular reactive oxygen species (ROS), leading to oxidative stress, DNA damage, and apoptosis, thereby accelerating skin aging and carcinogenesis [11,12]. As a natural UV absorber and antioxidant, melanin effectively scavenges free radicals and alleviates UVB-induced damage. Moreover, heavy metal ions such as lead and cadmium can enter the body through the food chain and trigger oxidative stress [13,14]; the metal-chelating capacity of melanin enables it to bind these ions and mitigate their toxicity [15,16].

In this study, melanin was extracted and purified from the fermentation broth of *O. sinensis* and further separated by column chromatography into two fractions, designated TZ-a and TZ-b. The structural heterogeneity of the two fractions was characterized by infrared spectroscopy, scanning electron microscopy, and thermogravimetric analysis. Furthermore, their photoprotective effects under UVB irradiation, protective effects against cell injury induced by four heavy metal ions (Cd^2+^, Pb^2+^, As^3+^, Hg^2+^). This study aims to provide a theoretical basis for the application of *O. sinensis* melanin in functional foods, cosmetics, and environmental remediation.

## 2. Materials and Methods

### 2.1. Materials

*Ophiocordyceps sinensis* fungus TZ8-1 is currently preserved in the Laboratory of Special Edible and Medicinal Fungi of Lanzhou Jiaotong University (Lanzhou, China).

The TZ8-1 strain was initially inoculated into a milk-based medium and incubated at 16 °C for 60 days. Subsequently, it was transferred to a liquid medium [7] and further cultivated at 18 °C on a rotary shaker (160 rpm) for another 60 days. After the cultivation period, TZ8-1 melanin was extracted from the filtered fermentation broth.

### 2.2. Extraction and Isolation of Melanin

The pH of the fermentation filtrate (1 L) was adjusted to approximately 14 with 1 mol/L NaOH solution, followed by magnetic stirring at 100 °C for 12 h. After centrifugation at 10,000× *g* for 30 min using an Eppendorf Centrifuge 5418R (Eppendorf AG, Hamburg, Germany), the pH of the collected supernatant was adjusted to 2.0 with 6 mol/L HCl solution. The mixture was then heated in a water bath at 100 °C for 12 h. The resulting black precipitate was collected by centrifugation, washed three times with distilled water, and freeze-dried to obtain the crude extract. Subsequently, the precipitate was sequentially washed with ethanol, chloroform, and ethyl acetate to remove soluble impurities. After washing with distilled water, the sample was freeze-dried to yield the TZ8-1 melanin sample [7]. Then, the TZ8-1 melanin sample from *O. sinensis* was dissolved in 88% formic acid and subjected to separation and purification by silica gel column chromatography (glass column 46 × 457 mm, 200–300 mesh silica gel, Qingdao Marine Chemical Co., Ltd., Qingdao, Shandong, China). The elution system consisted of a gradient sequence: methanol was used initially to elute small-molecular compounds (appearing as a pale yellow band). Subsequently, a methanol/formic acid mixture (1:2, *v*/*v*) was applied to elute TZ-a, which appeared as a deep brown band on the column. Then, rapid elution with formic acid yielded TZ-b, which appeared as a black band. Based on the differences in retention times and the distinct color contrast between TZ-a and TZ-b on the silica gel column, we initially separated the melanin into two components and concentrated using a RE-52AA Rotary Evaporator (Shanghai Yarong Biochemical Instrument Factory, Shanghai, China). Finally, 48 mg of TZ-a and 31 mg of TZ-b were obtained and stored at –20 °C until further analysis [17].

### 2.3. Physicochemical Characterization

#### 2.3.1. Field Emission Scanning Electron Microscopy (SEM)

Appropriate amounts of dried TZ-a and TZ-b powders were separately adhered onto conductive carbon tape, and non-adhering floating samples were blown off using a rubber suction bulb. The samples were then sputter-coated with gold under vacuum for 9 min. After coating, the samples were imaged using a field emission scanning electron microscope (ZEISS Gemini SEM 500, Carl Zeiss AG, Oberkochen, Baden-Württemberg, Germany) operated at an accelerating voltage of 5.0 kV. The particle morphology, surface structure, and distribution of melanin were examined and recorded at different magnifications.

#### 2.3.2. Fourier Transform Infrared Spectroscopy (FT-IR)

The structural characterization of melanin was performed using the KBr pellet method. Dried TZ-a and TZ-b powders (1 mg) were accurately weighed and mixed with 100 mg of spectral grade KBr powder in an agate mortar. The mixture was thoroughly ground under an infrared lamp to ensure homogeneity. The resulting mixture was transferred into a pellet die and pressed at 20 MPa for 1–2 min to form a transparent pellet. The pellet was then placed on the sample holder of a Fourier transform infrared spectrometer (VERTEX 70, Bruker Optik GmbH, Ettlingen, Baden-Württemberg, Germany). A pure KBr pellet was used as the background blank. Infrared spectra were recorded in the wavenumber range of 4000–400 cm^−1^, with 32 scans at a resolution of 4 cm^−1^. The infrared absorption spectrum of melanin was recorded, and the functional groups corresponding to the characteristic absorption peaks were analyzed.

#### 2.3.3. Thermogravimetric (TGA)

Thermogravimetric analysis was performed using a DTG-60AH Simultaneous Thermogravimetric-Differential Thermal Analyzer (Shimadzu Corporation, Kyoto, Japan). Approximately 2.5 mg of the sample was placed in an alumina crucible and measured under a high-purity nitrogen atmosphere at a gas flow rate of 50 mL/min. The temperature program was set as follows: starting from 30 °C, the temperature was increased to 700 °C at a heating rate of 10 °C/min, and the measurement was terminated at 700 °C. The thermogravimetric (TG) curve, showing the change in sample mass as a function of temperature, was recorded to analyze the thermal stability and thermal degradation behavior of the sample.

#### 2.3.4. Ultraviolet–Visible (UV–Vis) Spectroscopy

TZ-a and TZ-b were analyzed by UV–Vis spectrophotometry using a UV-2600 UV-Visible Spectrophotometer (Shimadzu Corporation, Kyoto, Japan) to determine their characteristic absorption peaks and λ max. Samples (1 mg) were dissolved in 2.0 mL of 0.1 mol/L NaOH, and spectra were recorded from 200 to 400 nm using the solvent as a blank control [7].

### 2.4. Research on Photoprotection Activity

#### 2.4.1. Cell Culture and Treatment

Human epidermal keratinocytes (NHEK, American Type Culture Collection (ATCC), Manassas, VA, USA) were cultured in serum-free medium (SFM, Absin Biotechnology Co., Ltd., Shanghai, China; Cat# abs9884) at 37 °C in a 5% CO_2_ atmosphere. The medium was changed every 2–3 days, and cells were passaged when they reached 80–90% confluence. Cells in the logarithmic growth phase were harvested by digestion with 0.25% trypsin-EDTA (1×, Gibco™, Thermo Fisher Scientific, Waltham, MA, USA), centrifuged, resuspended, and counted. Cell densities were adjusted according to the experimental requirements.

#### 2.4.2. Cell Viability Assay

NHEK cells were seeded in 96-well plates at a density of 5 × 10^3^ cells per well (100 μL per well in KGN serum-free medium (abs98843, Absin Bioscience Inc., Shanghai, China)) and pre-incubated at 37 °C in a 5% CO_2_ incubator for 24 h to allow cell attachment. Stock solutions of TZ-a and TZ-b were prepared by directly dissolving the solid samples in DMSO to ensure complete solubilization, with Vitamin C (VC, Sigma-Aldrich, St. Louis, MO, USA as the positive control. Both TZ-a and TZ-b were fully soluble at 200 μg/mL. Prior to UVB irradiation, the culture medium was aspirated, and the cells were gently washed twice with pre-warmed phosphate-buffered saline (PBS, Gibco™, Thermo Fisher Scientific, Waltham, MA, USA). A thin layer of PBS was then added to cover the cells to prevent desiccation during exposure. Irradiation was performed using a calibrated UVB light source at a distance of 15 cm for 83 s, delivering a total dose of 50 mJ/cm^2^. After irradiation, the PBS was removed, fresh medium was added, and the cells were incubated for an additional 24 h to allow recovery. To eliminate potential interference from melanin pigments with the absorbance measurements, the supernatant containing melanin was carefully aspirated, and the cells were gently washed once with PBS before adding 10 μL of CCK-8 reagent to each well. The plates were incubated for a further 4 h at 37 °C, and the absorbance was measured at 450 nm using Synergy H1 Microplate Reader (BioTek Instruments, Winooski, VT, USA). Cell viability was calcu Cell viability was measured using a Cell Counting Kit-8 (CCK-8, Beyotime Biotechnology, Haimen, Jiangsu, China; Cat# C0038) late according to Equation (1). This assay was used to assess the effects of UVB irradiation and compound pretreatment on cell viability.

Cell viability (%) = [(As − Ab)/(Ac − Ab)] × 100%(1)

#### 2.4.3. Measurement of Reactive Oxygen Species (ROS) Generation

The effect of TZ-a and TZ-b on intracellular reactive oxygen species (ROS) levels was evaluated using 2′,7′-dichlorodihydrofluorescein diacetate (DCFH-DA, Beyotime Biotechnology, Haimen, Jiangsu, China; Cat# S0033S). After UV-B irradiation and treatment with the test compounds, cells cultured in 35 mm dishes were incubated with 1 mL of 10 μM DCFH-DA probe in serum-free medium in the dark at 37 °C for 20–30 min. Subsequently, cells were washed three times with serum-free medium (SFM) to remove unincorporated probes. For nuclear counterstaining, Hoechst 33342 (Beyotime Biotechnology, Haimen, Jiangsu, China; Cat# C1022) was added to the cells at a final concentration of 1–5 μg/mL and co-incubated for 10–15 min. Finally, cells were washed again with PBS. Fluorescence images were immediately captured using an IX83 Inverted Fluorescence Microscope (Olympus Corporation, Tokyo, Japan). The excitation/emission wavelengths were set as follows: Ex = 488 nm/Em = 525 nm for DCF (green fluorescence indicating ROS), and Ex = 405 nm/Em = 461 nm for Hoechst (blue fluorescence indicating nuclei). All images were acquired with consistent exposure time and light intensity across all experimental groups. Subsequently, quantitative analysis of the fluorescence intensity was performed using ImageJ (Version 1.54f, National Institutes of Health, Bethesda, MD, USA) to determine relative ROS levels.

#### 2.4.4. Apoptosis Assay

The effect of TZ-a and TZ-b on cell apoptosis was evaluated by flow cytometry using Annexin V-FITC/PI double staining with an Annexin V-FITC/PI Apoptosis Detection Kit (Beyotime Biotechnology, Haimen, Jiangsu, China; Cat# C1062). Both supernatant and adherent cells were collected, washed with PBS, and digested with 0.25% trypsin-EDTA at 37 °C for 2–3 min. Complete medium was added to terminate digestion, and the cells were centrifuged. The supernatant was discarded, and the cells were resuspended and washed with PBS. The cells were then resuspended in 100 μL of 1 × Binding Buffer, followed by the addition of 5 μL of Annexin V-FITC and 5 μL of PI. The mixture was incubated for 15 min at room temperature in the dark, and then 400 μL of 1 × Binding Buffer was added. After filtration through a 40 μm cell strainer, at least 10,000 cells were collected for analysis. Flow cytometry was performed on a BD FACSCanto™ II Flow Cytometer (BD Biosciences, San Jose, CA, USA) with excitation at 488 nm, and fluorescence signals were detected at 530 nm (FITC) and above 670 nm (PI). Quantification of early and late apoptosis rates was performed using FlowJo (Version 10.9.0), with data visualization and statistical comparison conducted via GraphPad Software, Version 10.0.).

### 2.5. Heavy Metal Mitigation Capacity

#### 2.5.1. Cell Culture and Treatment

Cell culture was performed as described in Section 2.4.1. For CCK-8 and MDA/GSH assays, cells were seeded at 5 × 10^3^ cells/well (96-well plates) and 2 × 10^5^ cells/well (6-well plates), respectively, and allowed to attach for 24 h. Working concentrations of heavy metals were determined based on IC50 values pre-established via pilot experiments against NHEK cells. Stock solutions (10 mM) of arsenic trichloride (AsCl_3_, Sigma-Aldrich, St. Louis, MO, USA), cadmium chloride (CdCl_2_, Sigma-Aldrich, St. Louis, MO, USA; Cat# 202908), lead chloride (PbCl_2_, Sigma-Aldrich, St. Louis, MO, USA; Cat# 268690), and mercuric chloride (HgCl_2_, Sigma-Aldrich, St. Louis, MO, USA) were prepared in dilute HCl, sterilized through a 0.22 μm filter, and stored at 4 °C. Working solutions were freshly prepared by diluting stocks in complete medium, ensuring the final HCl concentration was ≤0.1% (*v*/*v*). The experimental groups were as follows: blank group (medium only), control group (cells only), model group (medium containing Pb^2+^/Cd^2+^/As^3+^/Hg^2+^), normal control group (Pb^2+^/Cd^2+^/As^3+^/Hg^2+^ + EDTA), and treatment groups (Pb^2+^/Cd^2+^/As^3+^/Hg^2+^ + different concentrations of TZ-a and TZ-b). Subsequently, cells were incubated in the dark for 24 h before further analysis.

#### 2.5.2. Cell Viability Assay

The cell viability was assessed using the CCK-8 assay. After the incubation period, 10 μL of CCK-8 solution was added to each well. The plates were gently shaken to ensure thorough mixing and then incubated in the dark at 37 °C for 4 h. The optical density (OD) of each well was measured at 450 nm using a microplate reader, and the cell viability was calculated according to Equation (1).

#### 2.5.3. Measurement of Malondialdehyde (MDA) Content

Intracellular malondialdehyde (MDA) content was determined using the thiobarbituric acid (TBA) method with a Beyotime lipid peroxidation MDA assay kit (Beyotime Biotechnology, Haimen, Jiangsu, China; Cat# S0131S). After treatment of cells in 6-well plates, the cells were washed twice with ice-cold PBS. Lysis buffer containing 1% protease inhibitor was added to each well to lyse the cells. The lysates were sonicated on ice using a JY92-IIN Ultrasonic Cell Crusher (Ningbo Xinzhi Biotechnology Co., Ltd., Ningbo, Zhejiang, China) and then centrifuged at 12,000 *g* for 10 min at 4 °C, and the supernatants were collected. An aliquot of the supernatant was used to determine the protein concentration using a BCA protein assay kit (Beyotime Biotechnology, Haimen, Jiangsu, China; Cat# P0012) for normalization of MDA content. Another aliquot (100 μL) of the supernatant was mixed with 200 μL of MDA detection working solution, heated in a boiling water bath for 15 min, and then cooled to room temperature under running water. The mixture was transferred to a 96-well plate, centrifuged, and the absorbance of the supernatant was measured at 532 nm using a microplate reader. MDA content was calculated according to a standard curve.

#### 2.5.4. Measurement of Glutathione (GSH) Content

Intracellular glutathione (GSH) content was determined using the DTNB method with a Beyotime assay kit (Beyotime Biotechnology, Haimen, Jiangsu, China; Cat# S0053. After treatment, cells were washed twice with ice-cold PBS and lysed in lysis buffer containing 1% protease inhibitor. The lysates were sonicated on ice and centrifuged at 12,000 *g* for 10 min at 4 °C, and the supernatants were collected. An appropriate volume of supernatant (adjusted to 100 μL with GSH assay buffer) was mixed with 150 μL of freshly prepared substrate working solution containing DTNB. After thorough mixing, the reaction was allowed to proceed for 5–25 min at room temperature. Absorbance was measured at 412 nm using a microplate reader, and total GSH content was calculated according to a standard curve.

### 2.6. Data Analysis

All experiments were performed in ≥3 independent replicates, with data presented as mean ± SD. Multiple-group comparisons used one-way ANOVA with Tukey’s post hoc test. Intergroup differences were annotated with lowercase letters: identical letters indicate no significance (p > 0.05), distinct letters denote significance (p < 0.05). Statistics were analyzed via SPSS Statistics (Version 27.0, IBM Corporation, Armonk, NY, USA) and GraphPad Prism (Version 10.0.0, GraphPad Software, Boston, MA, USA). Figures were generated using OriginPro (Version 2024, OriginLab Corporation, Northampton, MA, USA).

## 3. Results and Discussion

### 3.1. Physical and Chemical Characterization Analysis

#### 3.1.1. SEM Analysis

**Figure 1:**
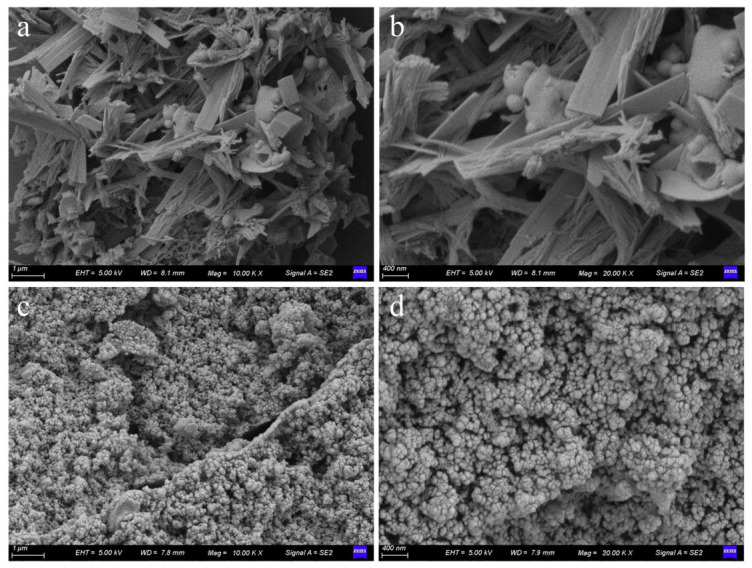
SEM images of TZ-a (**a**,**b**) and TZ-b (**c**,**d**) at 10,000× (**a**,**c**) and 20,000× (**b**,**d**).

SEM images (Figure 1) reveal marked morphological differences between the two fractions of melanin after column chromatography. TZ-a (Figure 1a,c) predominantly exhibits a lamellar or needle-like crystal morphology [18,19], with good dispersibility and a bundled or scattered distribution. In contrast, TZ-b (Figure 1b,d) forms larger block-like aggregates [20,21], with blurred particle boundaries and pronounced fusion and stacking. Under higher magnification (Figure 1d), TZ-b displays a dense and rough surface [22,23]. These observations indicate that TZ-a and TZ-b possess distinct morphological characteristics.

#### 3.1.2. FT-IR Analysis

**Figure 2:**
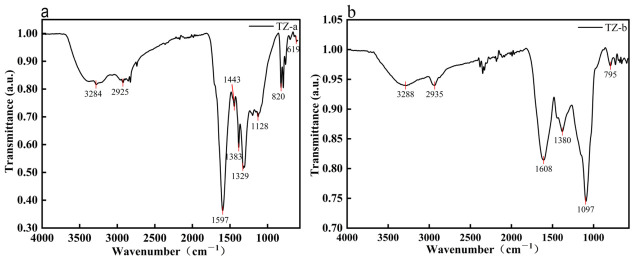
FT-IR spectra of TZ-a (**a**) and TZ-b (**b**).

The FT-IR spectrum of TZ-a (Figure 2a) exhibits characteristic absorption bands at 3248, 2925, 1597, 1443, 1383, 1329, 1128, 820,and 619 cm^−1^. The broad band at 3248 cm^−1^ is attributed to the stretching vibrations of O–H and N–H groups [23,24]. The absorption at 1597 cm^−1^ corresponds to aromatic C=C and/or C=O stretching vibrations [20,23]. The band at 1383 and 1329 cm^−1^ likely arises from the C–H bending vibration of the methyl group, the N-H bending of the amide group, and the C-N stretching [21,23]. The peak at 1128 cm^−1^ is assigned to the symmetric contraction of C–O–C or aliphatic C–O stretching [23]. The weak band at 820 cm^−1^ originates from the out-of-plane bending vibration of aromatic C–H groups [22,24]. Finally, the weak absorption peak at 600–700 cm^−1^ may be attributed to the C–S stretching vibration [9]. These peaks collectively indicated that TZ-a possessed the typical aromatic structure and amide groups of melanin.

The FT-IR spectrum of TZ-b (Figure 2b) shows absorption bands at 3282, 2935, 1608, 1097 and 795 cm^−1^. The broad band at 3282 cm^−1^ is assigned to O–H and N–H stretching vibrations [23,24]. The peak at 2935 cm^−1^ corresponds to aliphatic C–H stretching [22]. The band at 1608 cm^−1^ arises from aromatic C=C and/or C=O stretching [20,23]. The absorption at 795 cm^−1^ is due to the out-of-plane bending vibration of aromatic C–H groups [22]. The peak at 1097 cm^−1^ is attributed to the symmetric contraction of C–O–C or C–O stretching [23]. In contrast to TZ-a, TZ-b retains a complete aromatic skeleton and aliphatic side chains. In summary, both TZ-a and TZ-b exhibit the typical aromatic skeleton and functional group characteristics of melanin, but TZ-a contains amide groups, whereas TZ-b retains a complete aromatic skeleton and aliphatic side chains, indicating the structural differences between the two fractions.

#### 3.1.3. TGA

**Figure 3:**
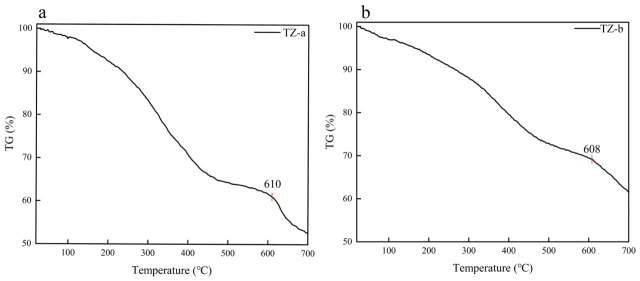
Thermogravimetric analysis diagrams of TZ-a (**a**) and TZ-b (**b**).

The thermogravimetric analysis results of the two fractions (TZ-a and TZ-b) are shown in Figure 3a,b. Throughout the temperature ramp from room temperature to 700 °C, both samples exhibit a continuous mass loss. At the initial stage (28 °C), both have a mass of 100%. Upon heating to 100 °C, the mass of both TZ-a and TZ-b decreases by approximately 3%, which is attributed to the evaporation of weakly bound water in the early stage [23]. In the temperature range of 200–400 °C, a significant mass loss occurs, presumably due to the disruption of the higher-order structure and substructure of melanin, the cleavage of non-covalent bonds between structural unit layers, and the breakage of covalent bonds within monomeric units [25]. At 500 °C, the mass losses of TZ-a and TZ-b reach 36% and 28%, respectively; at 700 °C, the mass losses are 48% and 39%, respectively. These data indicate that both TZ-a and TZ-b possess good thermal stability, and that TZ-b exhibits better thermal stability than TZ-a. Similar thermal stability patterns have been reported for bacterial melanin from Bacillus subtilis and Klebsiella sp., which also exhibited multi-stage weight loss profiles with major decomposition occurring above 500 °C [26,27].

#### 3.1.4. UV-Vis Analysis

**Figure 4:**
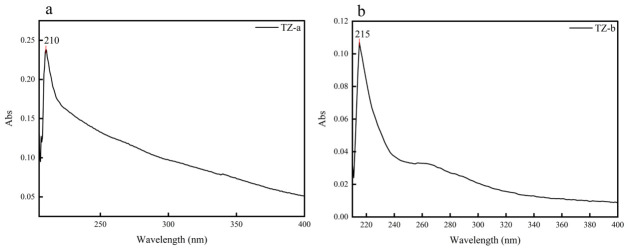
UV-Vis absorption spectra of TZ-a (**a**) and TZ-b (**b**) in 0.1 mol/L NaOH (0.5 mg/mL). Spectra were recorded from 200 to 400 nm using solvent as a blank. The full-range data are presented here; for the truncated spectra below 220 nm (omitted due to strong solvent absorption), see Supplementary Figure S1.

UV-Vis spectra of TZ-a and TZ-b melanin reveal that both melanins exhibit strong optical absorption in the UV region (Figure 4). The maximum absorption peak of TZ-a appears at 210 nm, while that of TZ-b appears at 215 nm, which is consistent with the absorption characteristics of many natural melanins [7,18,20,21].

### 3.2. Analysis of Photoprotection Activity

#### 3.2.1. Cell Survival Rate

**Figure 5:**
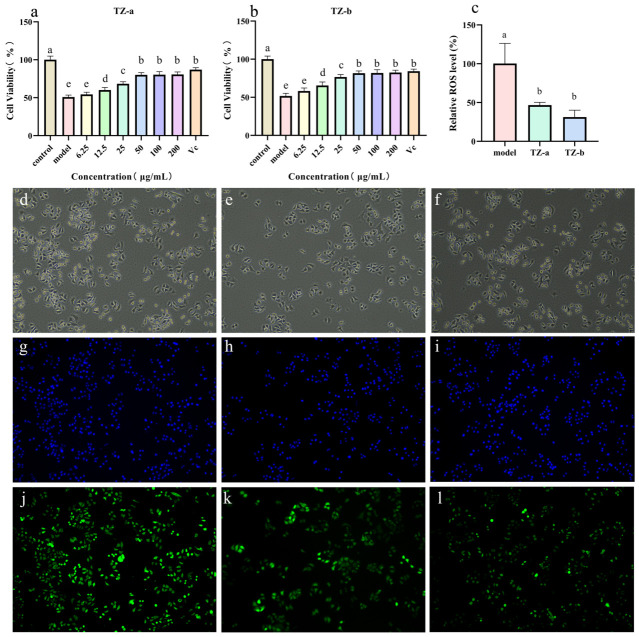
Cytoprotective and antioxidant effects of TZ-a and TZ-b against UVB irradiation. (**a**,**b**) Cell viability determined by CCK-8 assay. Among them, vitamin C (VC) was used as the positive control. (**c**) Quantitative analysis of relative ROS levels via DCFH-DA fluorescence. (**d**–**f**) Bright-field microscopy images: (**d**) Control (DGJ), (**e**) TZ-a treated (EHK), and (**f**) TZ-b treated (FIL). (**g**–**i**) DAPI staining of nuclei; (**j**–**l**) Corresponding ROS fluorescence (green). Data are presented as mean ± SD from three independent experiments (*n* = 3). Different lowercase letters (a–c) indicate significant differences among groups (*p* < 0.05).

The cytoprotective effects of TZ-a and TZ-b against UVB-induced damage were first evaluated by assessing cell viability. As shown in Figure 5a,b, both compounds increased cell viability in a concentration-dependent manner under UVB irradiation, which is consistent with previously reported protective actions of melanin-like polymers [28,29,30]. At a concentration of 200 μg/mL, TZ-a and TZ-b led to cell survival rates of 80 ± 3% and 82 ± 3%, respectively, indicating comparable protective capacities. Notably, these values are higher than those reported for certain natural phenols at similar concentrations [21], suggesting that TZ-a and TZ-b possess potent photoprotective potential. The slight difference between TZ-a and TZ-b may reflect subtle structural variations affecting their interaction with cellular membranes or melanin-uptake efficiency, an aspect that warrants further investigation. Similar photoprotective effects have been reported for melanin from *Lachnum* YM404, which significantly increased the survival of *E. coli*, *S. aureus*, and *S. cerevisiae* under UVB irradiation and alleviated UVB-induced skin damage in mice by enhancing SOD and GSH-Px activities while reducing MDA levels [31,32,33].

#### 3.2.2. Reactive Oxygen Species (ROS) Generation

The effect of TZ-a and TZ-b on intracellular reactive oxygen species (ROS) levels was assessed using the DCFH-DA fluorescent probe (Figure 5).

Given that melanin possesses the capacity to scavenge ROS and neutralize oxidative stress [8,20], this study evaluated their efficacy in mitigating UVB-induced oxidative stress. In the model group, relative ROS levels were significantly elevated (set as 100% for normalization), confirming the successful establishment of the oxidative stress model. Pre-treatment with 200 μg/mL of either TZ-a or TZ-b markedly attenuated the UVB-induced ROS accumulation. Specifically, TZ-a reduced the ROS level to approximately 46 ± 3%, whereas TZ-b exhibited a slightly stronger inhibitory effect, lowering it to around 31 ± 8%. Notably, the TZ-a and TZ-b groups showed significant differences compared to the model group (*p* < 0.05). The ROS levels in both the TZ-a and TZ-b treatment groups were significantly lower than that in the model group, confirming their potent ROS-scavenging activities. Although the difference between TZ-a and TZ-b did not reach statistical significance, this trend suggests that TZ-b may possess marginally superior radical-quenching capacity. This enhanced activity might be attributed to a higher density of phenolic hydroxyl groups or a more extended conjugated system in TZ-b [34]. While comparable ROS reductions have been reported for DHICA-melanin in UVA-irradiated HaCaT cells [1] the effective concentration of TZ-a/b appears higher, potentially reflecting differences in cellular uptake efficiency or the specific UVB irradiation protocol employed.

#### 3.2.3. Apoptosis

**Figure 6:**
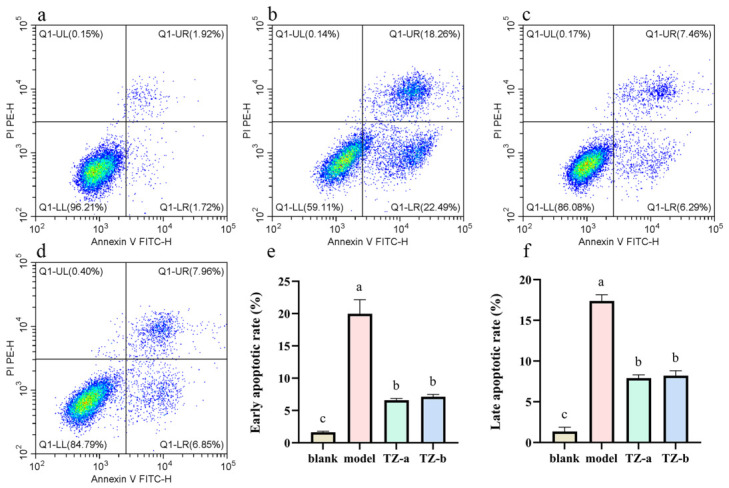
TZ-a and TZ-b attenuate UVB-induced apoptosis in NHEK cells. (**a**–**d**) Flow cytometric analysis of apoptosis via Annexin V-FITC/PI double staining: (**a**) blank control, (**b**) UVB model group, (**c**) TZ-a treated group, and (**d**) TZ-b treated group. Q1-UR: late apoptosis; Q1-LR: early apoptosis; Q1-LL: live cells; Q1-UL: necrosis. (**e**,**f**) Quantitative analysis of early (**e**) and late (**f**) apoptosis rates. Data are presented as mean ± SD (*n* = 3). Different lowercase letters indicate significant differences among groups (*p* < 0.05).

The extent of apoptosis in corneal tissues is shown in Figure 6. In the non-irradiated control group, early apoptotic (Q1-LR) and late apoptotic/necrotic (Q1-UR) cells accounted for approximately 1–2%, whereas viable cells (Q1-LL) represented 96–97%. UV-B irradiation alone (model group) significantly increased both early and late apoptotic fractions to about 16–18% each, and reduced viable cells to 59–64%, confirming that UV-B accelerates the apoptotic process. Treatment with 200 μg/mL of either TZ-a or TZ-b decreased early and late apoptotic populations to approximately 7–8% and restored viable cells to 84–86%. Compared to the model group, TZ-a and TZ-b increased the proportion of viable cells by about 27%. As quantified in panels (e) and (f), the early and late apoptosis rates in the UVB model group were 22.49% and 18.26%, respectively. Following treatment with TZ-a and TZ-b, these rates were significantly reduced to 6.85% and 7.96% (early), and 6.29% and 7.46% (late), respectively, aligning with the observed preservation of viable cells. These results demonstrate that both TZ-a and TZ-b effectively protect keratinocytes from UV-B-induced apoptosis, consistent with previous reports on melanin-based photoprotective agents [35,36,37,38]. The observed anti-apoptotic effect is likely mediated, at least in part, by the reduction in ROS levels, as demonstrated for other melanin polymers [1]. The complete graph of cell apoptosis can be found in Supplementary Material Figure S2.

### 3.3. Analysis of Heavy Metal Mitigation Capacity

#### 3.3.1. Cell Survival Rate

**Figure 7:**
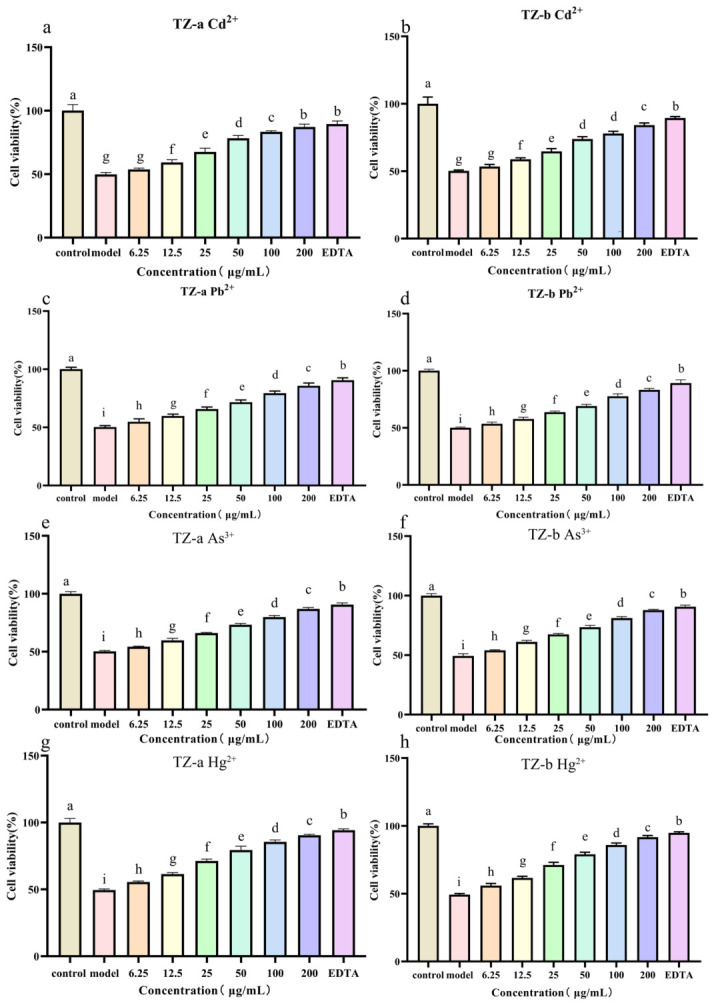
Mitigation of heavy metal toxicity by TZ-a and TZ-b in NHEK cells. (**a**–**h**) Effects of TZ-a or TZ-b pretreatment on cell viability under Cd^2+^, Pb^2+^, As^3+^, or Hg^2+^ exposure. Values represent mean ± SD (*n* = 3). Groups sharing different letters are significantly different (*p* < 0.05). IC50 values: Cd^2+^ = 11.05μmol/L; Pb^2+^ = 100.20μmol/L; As^3+^ = 8.97μmol/L; Hg^2+^ = 2.01μmol/L.

The cytoprotective effects of TZ-a and TZ-b against Cd^2+^, Pb^2+^, As^3+^, and Hg^2+^-induced cytotoxicity were evaluated by cell viability assay (Figure 7). Exposure to each heavy metal significantly reduced cell viability in the model group, with the calculated IC50 values being 11.05, 100.20, 8.97, and 2.01μmol/L for Cd^2+^, Pb^2+^, As^3+^, and Hg^2+^, respectively, confirming that heavy metals induce cell death [35,36,39,40]. Pre-incubation with TZ-a or TZ-b at concentrations ranging from 6.25 to 200 μg/mL increased cell viability in a concentration-dependent manner. At the highest concentration (200 μg/mL), the viability values for TZ-a and TZ-b were 87 ± 2% and 84 ± 1% under Cd^2+^, 85 ± 2% and 83 ± 1% under Pb^2+^ exposure, 86 ± 1% and 87 ± 1% under As^3+^, and 90 ± 1% and 91 ± 1% under Hg^2+^, respectively. Notably, the most pronounced protective effect was observed against Hg^2+^-induced toxicity for both compounds. These results indicate that TZ-a and TZ-b effectively mitigate heavy metal-induced cytotoxicity, likely through their metal-chelating and radical-scavenging properties as previously reported for melanin-related polymers [29,30]. The ability of melanin to interact with metal ions is well documented. Previous studies have shown that melanin from Inonotus hispidus exhibits varying stability in the presence of different metal ions, with Cu^2+^ exerting the most pronounced effect due to complexation with neighboring phenolic hydroxyl groups [38]. Similarly, melanin from *Rhizobium radiobacter* showed significant interactions with Fe^2+^ and Al^3+^, leading to flocculation and precipitation [23].

#### 3.3.2. MDA Content

**Figure 8:**
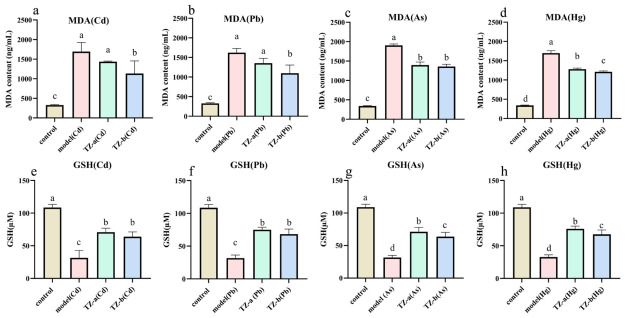
TZ-a and TZ-b mitigate heavy metal-induced oxidative stress in NHEK cells. (**a**–**d**) Malondialdehyde (MDA) levels indicating lipid peroxidation after exposure to Cd^2+^, Pb^2+^, As^3+^, or Hg^2+^. (**e**–**h**) Glutathione (GSH) levels reflecting the antioxidant capacity following treatment with TZ-a or TZ-b. Data are expressed as mean ± SD (*n* = 3). Different lowercase letters denote statistical significance (*p* < 0.05).

The effect of TZ-a and TZ-b on heavy metal-induced lipid peroxidation was assessed by measuring malondialdehyde (MDA) levels (Figure 8a–d). Compared with the blank control, the model groups exposed to Cd^2+^, Pb^2+^, As^3+^, or Hg^2+^ exhibited a marked increase in MDA content, indicating that heavy metal exposure induces significant oxidative stress [12,13,18,40]. Treatment with 200 μg/mL TZ-a or TZ-b substantially reduced intracellular MDA levels. Interestingly, TZ-b consistently showed greater inhibitory activity against MDA production than TZ-a across all four heavy metals, with the most pronounced difference observed in the Cd^2+^-exposed group. This differential effect may be attributed to structural differences between TZ-a and TZ-b that affect their radical-scavenging efficiency or cellular uptake [41], a hypothesis that warrants further investigation. Taken together, these data demonstrate that both compounds alleviate heavy metal-induced lipid peroxidation, with TZ-b exhibiting superior antioxidant potency under the tested conditions.

#### 3.3.3. GSH Content

The effect of TZ-a and TZ-b on intracellular glutathione (GSH) levels following heavy metal exposure is shown in Figure 8e–h. In all model groups, GSH levels were significantly decreased compared with the blank control, confirming that heavy metals deplete the cellular antioxidant reservoir [13,14,42,43]. Treatment with 200 μg/mL TZ-a or TZ-b restored GSH levels to near-control values. Combined with the MDA results (Figure 8), these findings suggest that the protective effects of TZ-a and TZ-b against heavy metal-induced oxidative damage are mediated, at least in part, through the preservation of intracellular GSH pools and the inhibition of lipid peroxidation. Notably, TZ-a was slightly superior to TZ-b in restoring GSH levels, whereas TZ-b exhibited stronger activity in reducing MDA content, particularly under Cd^2+^ stress, suggesting that the two compounds may exert synergistic antioxidant effects through distinct mechanisms. The observed reduction in MDA and restoration of GSH levels by TZ-a and TZ-b are consistent with the well-established metal-chelating properties of melanin. Singh comprehensively reviewed the capacity of microbial melanin to bind heavy metals such as Cd^2+^, Cu^2+^, and Zn^2+^ through carboxyl, phenolic hydroxyl, and amino groups, highlighting its potential for bioremediation and cytoprotection [9]. A limitation of this study is that the direct metal-chelating capacity of TZ-a and TZ-b was not quantified; future work should address this to fully elucidate the structure–activity relationship.

## 4. Conclusions

In this study, two melanin fractions, TZ-a and TZ-b, were isolated from TZ8-1 melanin and characterized by FT-IR, SEM, TGA and UV-Vis. UV-Vis analysis confirmed the characteristic broadband UV absorption of both fractions, validating their identity as melanin pigments [20,21]. FT-IR analysis revealed that TZ-a exhibited absorption peaks at 600–700 cm^−1^ and 1443 cm^−1^, which were similar to the infrared spectra of melanin in the literature [27,28,29], indicating that it contains sulfur-containing units in its structure, while TZ-b showed typical spectral characteristics of true melanin. SEM showed well-dispersed lamellar or needle-like crystals for TZ-a, in contrast to large, fused block-like aggregates with rough surfaces for TZ-b, suggesting distinct molecular packing behaviors, and TGA demonstrated good thermal stability for both, with TZ-b being more stable than TZ-a. These structural, morphological, and thermal differences highlight the chemical heterogeneity of natural melanin and may underlie their distinct biological activities. In photoprotection assays, both compounds exhibited potent activity against UVB-induced damage in keratinocytes, increasing cell viability in a concentration-dependent manner to 80.59% and 82.48% at 200 μg/mL, respectively, suppressing intracellular ROS generation (with TZ-b showing superior scavenging activity), and reducing early and late apoptosis, thereby restoring viable cells to 80–82% [44,45]. Against heavy metal-induced cytotoxicity (Cd^2+^, Pb^2+^, As^3+^, Hg^2+^), both fractions showed concentration-dependent cytoprotective effects, most pronounced under Hg^2+^ stress, significantly lowering malondialdehyde levels and restoring depleted glutathione pools; notably, TZ-b was more effective in inhibiting MDA production while TZ-a was slightly better at restoring GSH levels, suggesting complementary antioxidant mechanisms. The heavy metal mitigation capacity of TZ-a and TZ-b is likely attributable to their ability to chelate metal ions via surface functional groups, as previously demonstrated for melanin from other microbial and fungal sources [9,23,38]. Collectively, these findings demonstrate that TZ-a and TZ-b possess potent antioxidant and cytoprotective properties, likely attributable to phenolic hydroxyl and carboxyl groups [46], metal chelation, and radical scavenging, and highlight their potential as effective ingredients in dermo-cosmetic or therapeutic formulations for the prevention of UVB-induced skin damage, heavy metal toxicity, and oxidative stress-related disorders. A limitation of this study is that direct metal-chelating capacities were not quantified; future investigations should address this to fully elucidate the structure–activity relationship.

## Data Availability

The data presented in this study are available on request from the corresponding author.

## Supplementary Materials

The following supporting information can be downloaded at: https://www.mdpi.com/article/10.3390/biology15141183/s1. Figure S1. Representative flow cytometry dot plots of apoptosis. Figure S2. UV-Vis absorption spectra of TZ-a and TZ-b in the 220–400 nm range.

## References

[1] Liberti D., Alfieri M.L., Monti D.M., Panzella L., Napolitano A. (2020). A Melanin-Related Phenolic Polymer with Potent Photoprotective and Antioxidant Activities for Dermo-Cosmetic Applications. Antioxidants.

[2] Shi F., Li J., Ye Z., Yang L., Chen T., Chen X., Ye M. (2018). Antitumor Effects of Melanin from *Lachnum* YM226 and Its Derivative in H22 Tumor-Bearing Mice†. RSC Med. Chem..

[3] Peng Z., Luo S., Zhao D., Zhang J. (2023). Biosynthetic Melanin with Excellent Performance Can Be Used for Heavy Metal Adsorption. J. Clean. Prod..

[4] Li G., Hu Q., Xing R., Zhang J., Chen Y. (2026). Metabolites of *Ophiocordyceps sinensis* from Two Typical Growing Regions in China Revealed by Comparative Metabolomics. J. Future Foods.

[5] Jia L.-F., Chen P., Qu G.-D., Sun N., Guo T., Zhong H.-Y., Duan Y.-H., Sun J.-H., Sun J.-B. (2025). A Comprehensive Review of the Sedative-Hypnotic Mechanisms of Edible Fungi. Food Med. Homol..

[6] Tan X.-Y., Jiang T., Hu Q.-C., Hai Y.-P., Lu X.-H., Jin C.-M., Ma X., Li Y.-Y., Efferth T. (2026). Drug-Induced Liver Injury Therapy: Unveiling the Healing Potential of Natural Products from Medicinal and Food Homology Plants. Food Med. Homol..

[7] Tong C., Luo J., Xie C., Wei J., Pan G., Zhou Z., Li C. (2023). Characterization and Biological Activities of Melanin from the Medicinal Fungi *Ophiocordyceps sinensis*. Int. J. Mol. Sci..

[8] Dong C., Yao Y. (2012). Isolation, Characterization of Melanin Derived from *Ophiocordyceps sinensis*, an *Entomogenous fungus* Endemic to the Tibetan Plateau. J. Biosci. Bioeng..

[9] Singh S., Nimse S.B., Mathew D.E., Dhimmar A., Sahastrabudhe H., Gajjar A., Ghadge V.A., Kumar P., Shinde P.B. (2021). Microbial Melanin: Recent Advances in Biosynthesis, Extraction, Characterization, and Applications. Biotechnol. Adv..

[10] Tran-Ly A.N., Reyes C., Schwarze F.W.M.R., Ribera J. (2020). Microbial Production of Melanin and Its Various Applications. World J. Microbiol. Biotechnol..

[11] Mohania D., Chandel S., Kumar P., Verma V., Digvijay K., Tripathi D., Choudhury K., Mitten S.K., Shah D. (2017). Ultraviolet Radiations: Skin Defense-Damage Mechanism. Adv. Exp. Med. Biol..

[12] Liu Y., Du C., Lin C., Gao X., Zhu J., Zhang C. (2022). Characterization of Copper/Zinc Superoxide Dismutase Activity on *Phascolosoma esculenta* (Sipuncula: *Phascolosomatidea*) and Its Protection from Oxidative Stress Induced by Cadmium. Int. J. Mol. Sci..

[13] Akpinar A., Cansev A. (2024). Choline Supplementation Reduces Cadmium Uptake and Alleviates Cadmium Toxicity in *Solanum lycopersicum* Seedlings. BMC Plant Biol..

[14] Ghouri F., Jin J., Ali S., Zhong M., Liu J., Xia W., Jin F., Shahid M.Q. (2025). Metabolomic, Biochemical, and Cytological Observations Reveal β-Pinene’s Protective Effects against Cadmium Toxicity in Salt-Tolerant Rice. J. Environ. Manag..

[15] Bothma J.P., Boor J.d., Divakar U., Schwenn P.E., Meredith P. (2008). Device-Quality Electrically Conducting Melanin Thin Films. Adv. Mater..

[16] Nosanchuk J.D., Stark R.E., Casadevall A. (2015). Fungal Melanin: What Do We Know About Structure?. Front. Microbiol..

[17] Rosas Á.L., Nosanchuk J.D., Gómez B.L., Edens W.A., Henson J.M., Casadevall A. (2000). Isolation and Serological Analyses of Fungal Melanins. J. Immunol. Methods.

[18] Wu C.-C., Li H., Yin Z.-W., Zhang H.-T., Gao M.-J., Zhu L., Zhan X.-B. (2022). Isolation, Purification, and Characterization of Novel Melanin from the Submerged Fermentation of *Rhizobium radiobacter*. Process Biochem..

[19] Oh J.-J., Kim J.Y., Kwon S.L., Hwang D.-H., Choi Y.-E., Kim G.-H. (2020). Pigments Derived from *Amorphotheca resinae*. J. Microbiol..

[20] Ye M., Wang Y., Guo G., He Y., Lu Y., Ye Y., Yang Q., Yang P. (2012). Physicochemical Characteristics and Antioxidant Activity of Arginine-Modified Melanin from *Lachnum* YM-346. Food Chem..

[21] Liu Q., Xiao J., Liu B., Zhuang Y., Sun L. (2018). Study on the Preparation and Chemical Structure Characterization of Melanin from *Boletus griseus*. Int. J. Mol. Sci..

[22] Kumar B.T.S., Rao B.V.S., Swathi S., Vanajakshi V., Hebbar H.U., Singh S.A. (2024). Characterization, Antioxidant, and Antimicrobial Activity of Melanin Extracted from Nigerseed Hulls. Food Biosci..

[23] Pralea I.-E., Moldovan R.-C., Petrache A.-M., Ilieș M., Hegheș S.-C., Ielciu I., Nicoară R., Moldovan M., Ene M., Radu M. (2019). From Extraction to Advanced Analytical Methods: The Challenges of Melanin Analysis. Int. J. Mol. Sci..

[24] Hou L., Yu J., Zhao L., He X. (2020). Dark Septate Endophytes Improve the Growth and the Tolerance of Medicago Sativa and *Ammopiptanthus mongolicus* under Cadmium Stress. Front. Microbiol..

[25] Xin C., Ma J., Tan C., Yang Z., Ye F., Long C., Ye S., Hou D. (2015). Preparation of Melanin from *Catharsius molossus* L. and Preliminary Study on Its Chemical Structure. J. Biosci. Bioeng..

[26] Gómez-Marín A.M., Sánchez C.I. (2010). Thermal and Mass Spectroscopic Characterization of a Sulphur-Containing Bacterial Melanin from *Bacillus subtilis*. J. Non-Cryst. Solids.

[27] Sajjan S.S. (2013). Properties and Functions of Melanin Pigment from *Klebsiella* sp. GSK. Korean J. Microbiol. Biotechnol..

[28] Goncalves R.D.C.R., Pombeiro-Sponchiado S.R. (2005). Antioxidant Activity of the Melanin Pigment Extracted from *Aspergillus nidulans*. Biol. Pharm. Bull..

[29] Pacelli C., Cassaro A., Maturilli A., Timperio A.M., Gevi F., Cavalazzi B., Stefan M., Ghica D., Onofri S. (2020). Multidisciplinary Characterization of Melanin Pigments from the Black Fungus *Cryomyces antarcticus*. Appl. Microbiol. Biotechnol..

[30] Lin L., Xu J. (2020). Fungal Pigments and Their Roles Associated with Human Health. J. Fungi.

[31] Ye M., Guo G., Lu Y., Song S., Wang H., Yang L. (2014). Purification, Structure and Anti-Radiation Activity of Melanin from *Lachnum* YM404. Int. J. Biol. Macromol..

[32] Paolo W.F., Dadachova E., Mandal P., Casadevall A., Szaniszlo P.J., Nosanchuk J.D. (2006). Effects of Disrupting the Polyketide Synthase Gene *WdPKS1* in *Wangiella* [Exophiala] *dermatitidis* on Melanin Production and Resistance to Killing by Antifungal Compounds, Enzymatic Degradation, and Extremes in Temperature. BMC Microbiol..

[33] Venil C.K., Velmurugan P., Dufossé L., Devi P.R., Ravi A.V. (2020). Fungal Pigments: Potential Coloring Compounds for Wide Ranging Applications in Textile Dyeing. J. Fungi.

[34] Panzella L., Gentile G., D’Errico G., Della Vecchia N.F., Errico M.E., Napolitano A., Carfagna C., d’Ischia M. (2013). Atypical Structural and π-Electron Features of a Melanin Polymer That Lead to Superior Free-Radical-Scavenging Properties. Angew. Chem..

[35] Suthar M., Dufossé L., Singh S.K. (2023). The Enigmatic World of Fungal Melanin: A Comprehensive Review. J. Fungi.

[36] Marcovici I., Coricovac D., Pinzaru I., Macasoi I.G., Popescu R., Chioibas R., Zupko I., Dehelean C.A. (2022). Melanin and Melanin-Functionalized Nanoparticles as Promising Tools in Cancer Research—A Review. Cancers.

[37] Schmaler-Ripcke J., Sugareva V., Gebhardt P., Winkler R., Kniemeyer O., Heinekamp T., Brakhage A.A. (2009). Production of Pyomelanin, a Second Type of Melanin, via the Tyrosine Degradation Pathway in *Aspergillus fumigatus*. Appl. Environ. Microbiol..

[38] Hou R., Liu X., Xiang K., Chen L., Wu X., Lin W., Zheng M., Fu J. (2019). Characterization of the Physicochemical Properties and Extraction Optimization of Natural Melanin from *Inonotus hispidus* Mushroom. Food Chem..

[39] Dadachova E., Casadevall A. (2008). Ionizing Radiation: How Fungi Cope, Adapt, and Exploit with the Help of Melanin. Curr. Opin. Microbiol..

[40] Solano F. (2014). Melanins: Skin Pigments and Much More—Types, Structural Models, Biological Functions, and Formation Routes. New J. Sci..

[41] Yang C.-J., Nguyen D.D., Lai J.-Y. (2023). Poly(l-Histidine)-Mediated On-Demand Therapeutic Delivery of Roughened Ceria Nanocages for Treatment of Chemical Eye Injury. Adv. Sci..

[42] Liu Q., Sun M., Wang T., Zhou Y., Sun M., Li H., Liu Y., Xu A. (2023). The Differential Antagonis tic Ability of Curcumin against Cytotoxicity and Genotoxicity Induced by Distinct Heavy Metals. Toxics.

[43] Paladines-Beltrán G.M., Venegas N.A., Suárez J.C. (2025). Arbuscular Mycorrhizal Fungi Enhance Antioxidant Defense Systems in Sugarcane under Soil Cadmium Stress. Plants.

[44] Solano F. (2020). Photoprotection and Skin Pigmentation: Melanin-Related Molecules and Some Other New Agents Obtained from Natural Sources. Molecules.

[45] Suárez-Vergel G., Garcia-Ortiz N., Loera O., López-Pérez M. (2026). Water-soluble Cordyceps melanin: A photoprotector that en-hances the survival of Beauveria bassiana and Metarhizium acridum conidia under UV-B radiation. World J. Microb. Biot..

[46] Al-Shamery N., Biyashev D., Blancafort L., Camus A., Gianneschi N.C., De Olivera Graeff C.F., Kohler B., Li S., Lu K.Q., Lumb J.-P. (2025). From melanogenesis to melanin technologies. Commun. Chem..

