# Supplementary material for: Photoprotective and Heavy Metal-Detoxifying Melanin Fractions Isolated from Ophiocordyceps sinensis Fermentation Products
Source: Biology (Basel). 2026 Jul 17;15(14):1183. doi: 10.3390/biology15141183 (PMC13404823; doi:10.3390/biology15141183)
Supplement: Supplementary file 1 [file biology-15-01183-s001.zip › biology-4395005-supplementary.pdf]

The full apoptosis images are included in the supplementary material (Supplementary Figure S1).

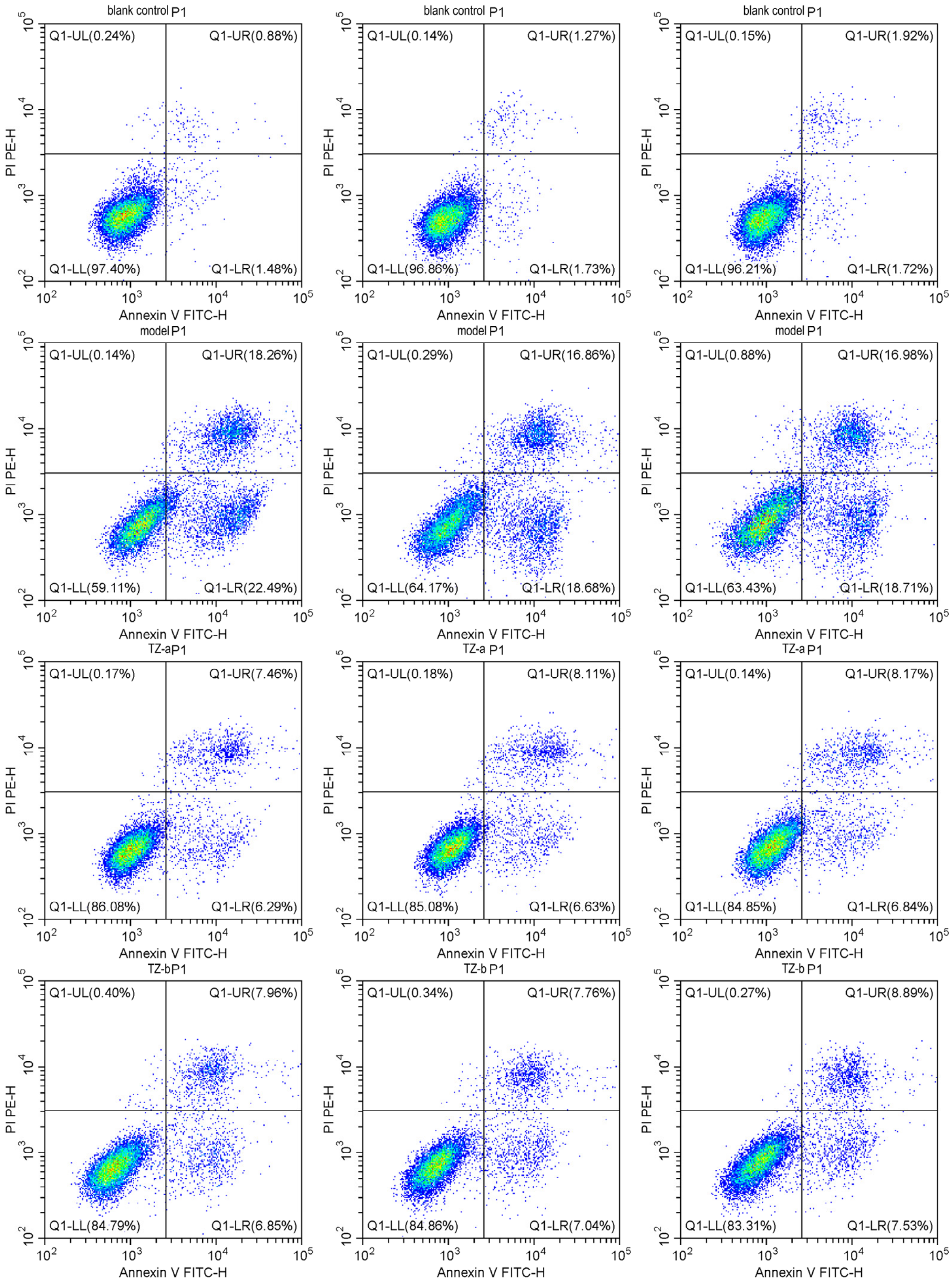

Supplementary Figure S1. Representative flow cytometry dot plots of apoptosis.

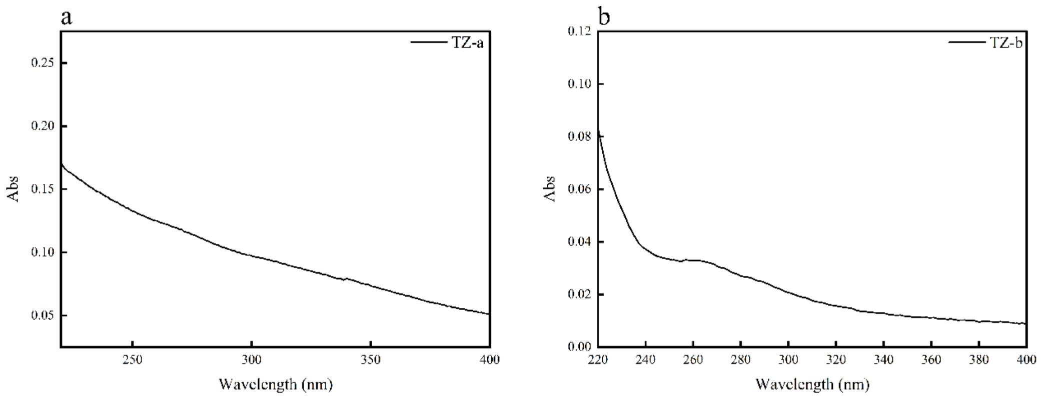

Supplementary Figure S2. UV-Vis absorption spectra of TZ-a and TZ-b in the 220–400 nm range.

Samples were dissolved in 0.1 mol/L NaOH at a concentration of 0.5 mg/mL. Both TZ-a and TZ-b exhibit broad absorbance bands in the 240–400 nm range, with TZ-b additionally showing a shoulder at 260 nm.
